# Association between Serum Uric Acid Levels and Sleep Variables: Results from the National Health and Nutrition Survey 2005–2008

**DOI:** 10.1155/2012/363054

**Published:** 2012-08-28

**Authors:** R. Constance Wiener, Anoop Shankar

**Affiliations:** ^1^Department of Dental Practice and Rural Health, Health Sciences North, West Virginia University, P.O. Box 9448, Morgantown, WV 26506, USA; ^2^Department of Epidemiology, School of Public Health, Health Sciences North, West Virginia University, P.O. Box 9190, Morgantown, WV 26506, USA

## Abstract

Sleep disordered breathing as well as high serum uric acid levels are independent risk factors for cardiovascular disease. However, studies evaluating the relationship between sleep-disordered breathing and hyperuricemia are limited. We examined the 2005–2008 National Health and Nutrition Examination survey's sleep variables and high serum uric acid among 6491 participants aged ≥20 years. The sleep variables included sleep duration, snoring, snorting, and daytime sleepiness. The main outcome was high serum uric acid level, defined as levels of serum uric acid >6.8 mg/dL in males and >6.0 mg/dL in females. We found that snoring more than 5 nights per week, daytime sleepiness, and an additive composite score of sleep variables were associated with high serum uric acid in the age- , sex-adjusted model and in a multivariable model adjusting for demographic and lifestyle/behavioral risk factors. The association was attenuated with the addition of variables related to clinical outcomes such as depression, diabetes, hypertension, and high-cholesterol levels. Our results indicate a positive relationship between sleep variables, including the presence of snoring, snorting, and daytime sleepiness, and high serum uric acid levels.

## 1. Introduction

Sleep-disordered breathing (SDB) is a spectrum of disruptive breathing patterns in sleep in which the upper airway is partially or completely obstructed resulting in airflow symptoms which may vary from mild to severe airflow changes, oxygen desaturation, and arousals [1, 2], or there may be central involvement (central sleep apnea) in which there is a lack or loss of the drive to breathe during sleep. Central sleep apnea occurs as a result of high altitude-induced periodic breathing, idiopathic CSA, narcotic-induced central apnea, obesity hypoventilation syndrome, and Cheyne-Stokes breathing [3]. SDB has been associated with hypertension, cardiovascular disease, and increased risk of fatal and nonfatal cardiovascular events [4–7]. 

Higher levels of serum uric acid have been shown to be associated with increased risk of hypertension, [8–14] prehypertension, [15] peripheral arterial disease, [16] diabetes mellitus [17], and cardiovascular disease through inflammatory pathways [4–7]. Animal studies have suggested that SDB is related to elevations in uric acid levels [1, 18]. The hypothesized mechanism is that during hypoxia, there is increased adenosine triphosphate degradation and the release of purine intermediates (adenosine, inosine, hypoxanthine, and xanthine) as well as the purine catabolic end product, and uric acid. [1, 18]. However, the association between SDB and high uric acid levels in humans is not clear. While some studies [18–21] have reported an association between SDB markers and high serum uric acid levels, others have not [22, 23]. Also, most of the previous studies had small sample sizes and did not examine the association by gender and race/ethnicity. In this context, we examined the association between sleep duration, snoring, snorting, and daytime sleepiness and high serum uric acid in a large, nationally representative sample of US adults, examining the confounding by age, gender, race/ethnicity, education, smoking status, alcohol intake, physical activity, body mass index, depression, diabetes, and total cholesterol. In this hypothesis generating study, our research hypothesis was that high levels of serum uric acid would be associated with the sleep variables of sleep duration, snoring, snorting, daytime sleepiness, and an additive summary score of the variables.

## 2. Methods

Data from the National Health and Nutrition Examination Survey (NHANES) 2005-2006 and 2007-08 were used in this study. The study design and study methodology of the NHANES are available in details elsewhere [24, 25]. In brief, the national survey is charged to monitor the health and dietary status of children and adults in the United States. The survey design utilizes stratified probability samples in multiple stages to develop an accurate representation of the noninstitutionalized civilian population [24]. NHANES oversamples non-Hispanic blacks and Mexican Americans to provide an adequate sample size to analyze the data based upon race/ethnicity. The methods employed in the survey included clinical, physical, and laboratory examinations as well as interviews. A sampling algorithm was used, taking into account counties, blocks, households, and individuals within a household [24]. 

 Of the 20497 participants in NHANES 2005-2006 and 2007-2008, there were 10914 who were age 20 years or older. We excluded participants who were pregnant (*n* = 393), had prevalent cardiovascular disease (*n* = 1275), had missing sleep variables (*n* = 1375), or had missing uric acid variables or other missing variables for our multivariable logistic regression models (*n* = 1380). Uric acid data in the laboratory portion of the NHANES survey was provided by individuals aged 12 years or older. The final analysis consisted of 6491 participants for whom complete multivariable information was available.

### 2.1. Outcome of Interest: High Serum Uric Acid

High serum uric acid was defined as serum levels of uric acid > 6.8 mg/dL in males based upon clinical usefulness, and previous research [26–28], as defining hyperuricemia is based on the concentration equal or are above the limit of urate solubility [28]. Likewise, high uric acid was defined as >6.0 mg/dL for females based upon previous research and clinical usefulness [29]. The laboratory collection procedure is available in the NHANES Laboratory Procedures Manual [24, 25].

### 2.2. Assessment of Exposure: Sleep Variables

Sleep variables were self-reported by the participants. The NHANES interview questions which regarded sleep behavior were “How much sleep do you usually get at night and on weekdays or workdays?”; “In the past 12 months, how often did you snore while you were sleeping?”; “In the past 12 months, how often did you snort, gasp, or stop breathing while you were asleep?”; “In the past month, how often did you feel excessively or overly sleepy during the day?” From the four sleep questions, we derived four sleep variables identified as sleep duration; snoring; snorting; daytime sleepiness. The 5 categories for sleep duration consisted of less than or equal to 5 hours of sleep, 6 hours of sleep, 7 hours of sleep, 8 hours of sleep, and greater than or equal to 9 hours of sleep. Our snorting, and snoring variables were categorized from the participants' responses of “never or rare” to the corresponding 0–2 times/week descriptor, “occasional” to 3-4 times/week, and “frequent” to 5 or more times/week. Our daytime sleepiness variable combined participants' responses of “never or rare,” “sometimes,” and “often” into the 0–15 times/month combination of those responses and “almost always,” using the 16 or more times/month descriptor.

An ad hoc additive composite score of the sleep variables was designed to study the simultaneous additive effect of the multiple sleep variables and was based upon previous research [30–32]. To create the additive composite score, we dichotomized the four sleep variables, based upon clinical significance, and then we combined them. Sleep duration was dichotomized into a category of 5 hours of sleep or less and a category of greater than 5 hours of sleep. Snoring was dichotomized into snoring 3-4 nights or more per week and snoring less than 3-4 nights per week. Snorting was dichotomized into snorting 3-4 nights or more per week and snorting less than 3-4 nights per week. The daytime sleepiness variable was created as a dichotomized variable with 16 times or more per month as the cut-point. Each positive category was assigned a score of 1. The individual scores were accumulated into the additive composite score of sleep variables, which therefore had a range of values from 0 to 4. The additive composite score 0, represented that no sleep variables were present; whereas a score of 4 indicated the presence of all 4 variables.

### 2.3. Assessment of Covariates

The covariates used from the NHANES survey were age, sex, race/ethnicity, education, smoking, alcohol intake, physical activity, body mass index, hypertension, depression, diabetes, and cholesterol. Self-report was the basis for age, sex, race/ethnicity, education, smoking, alcohol intake, depression, and diabetes. The questions posed for alcohol use asked the amount of alcohol intake in grams/day. Current alcohol use was defined as 1 or more alcoholic drinks in the previous year. The educational level was categorized as less than high school graduation, high school graduation, and more than high school graduation. Smoking status was defined as never use (less than 100/lifetime), current use (a history of 100 or more cigarettes and continued use), and former use (a history of 100 or more cigarettes and no longer smoking). Moderate physical activity was defined as activities which increased the heart and breathing rate such as walking briskly, cycling, swimming, or playing golf for at least 10 minutes per week. Depression was self-identified as feeling down, depressed, or hopeless more than half of the days or nearly every day within the previous two weeks. 

Physical and laboratory evaluations were conducted by the medical examination center (MEC). Body mass index (BMI) was derived as weight in kilograms divided by height in meters-squared. A mercury sphygmomanometer was used to take 3 blood pressure measurements and the mean systolic and diastolic were recorded. High blood pressure was defined as a blood pressure with a mean systolic of 140 mm Hg or above, or a mean diastolic blood pressure of 9 mm Hg or above, or a positive response to the use of blood pressure reducing medications.

Total serum cholesterol levels were determined enzymatically using the Roche Hitachi 717 in 2005, Roche Hitachi 717 and 912 in 2006, and the Roche Modular P chemistry analyzer in 2007-2008. The details of blood collection, processing, and analysis are available in the Laboratory Procedures Manuals [24, 25]. 

### 2.4. Statistical Analysis

Descriptive characteristics of the study participants by high serum uric acid (serum levels of uric acid > 6.8 mg/dL in males and > 6.0 mg/dL in females) were determined utilizing the chi-square test or analysis of variance, as needed. The association of the sleep variables (sleep duration, snoring, snorting, daytime sleepiness, and the adhoc additive composite score of sleep variables and serum uric acid were evaluated with three logistic regression models. We completed (1) an age-(years), gender-(male, female) adjusted logistic regression model; (2) a multivariable model 1 in which we adjusted for age (years), gender (male, female), race/ethnicity (non-Hispanic whites, non-Hispanic blacks, Mexican Americans, and others), education (below high school, high school, and above high school), smoking (never smoker, former smoker, and current smoker), alcohol intake (absent, present), moderate physical activity (absent, present) and body mass index (obese, nonobese) and (3) a multivariable model 2 in which we also included hypertension (absent, present), depression (absent, present), diabetes (absent, present), and total cholesterol (mg/dL). In these analyses, we used logistic regression models with the categorical high serum uric acid (level > 6.8 mg/dL in males, and > 6.0 mg/dL in females) as the outcome variable. Trend tests were performed with the sleep variables and the summary score as an ordinal variable in the corresponding multivariable models. Subgroup analysis by gender, and race/ethnicity were also conducted. To examine potential collinearity, regression diagnostics for collinearity were performed; there were no significant collinearity of variables included in the multivariable model. The analyses were weighted to adjust for unequal probabilities of selection, oversampling, and nonresponse by using SUDAAN (version 8.0; Research Triangle Institute, Research Triangle Park, NC) and SAS (version 9.1.; SAS Institute, Cary, NC) software.

## 3. Results

A tabular description of the study population based upon the additive composite score of sleep variables is presented in Table 1. There were more participants who had a composite score of sleep variables of zero who were educated above high school, who exercised moderately, who had lower cholesterol, who were normal weight, not depressed, and who did not have diabetes.

Overall, the study sample population was a middle-aged (mean age 44.8 years) multiethnic sample consisting of non-Hispanic whites (72.6%), non-Hispanic blacks (10.0%), and Mexican Americans and other race/ethnicities (17.4%). The majority (58.4%) of this sample was educated above the high school level, and consisted of current drinkers (74.3%). There was an equal distribution of normal weight, overweight, and obese BMI groups. Never smokers and former smokers comprised 76.4% of the sample, (52.8% and 23.6%, resp.), and current smokers comprised 23.6%. Also, depression was reported by 5.1% and diabetes mellitus was present in 8.9% of the subjects. The mean total cholesterol was 200.1 mg/dL.

Table 2 presents the associations between various sleep variables, including sleep duration, snoring, snorting, daytime sleepiness, and the additive composite score of sleep variables and their association with high serum uric acid, defined as serum levels > 6.8 mg/dL in males and 6.0 mg/dL in females. We found that sleep duration was significantly associated with high serum uric acid for the sleep duration of 8 hours, which was protective in each of the models.

The adjusted odds ratios for daytime sleepiness, and the additive composite score were associated with high serum uric acid in the age-, gender-adjusted model. The associations were maintained and had increased in multivariable model 1. The associations were attenuated for multivariable model 2. The adjusted odds ratios' confidence intervals for multivariable model 2 included the value of 1, indicating that the associations for multivariable model 2 may be due to chance. Similarly, the adjusted odds ratios for snorting had confidence intervals which included the value of 1, indicating that the associations of uric acid and snorting may be due to chance. 

The adjusted odds ratios for snoring 5 hours or more were significant across all models. 

In terms of trend, there was a significant *P*-trend for sleep duration, snoring, daytime sleepiness, and with the additive composite score of sleep variables across the age-, sex-adjusted and multivariable 1 logistic regression models. The trend continued with significance for snoring in the multivariable model 2.

Table 3 presents the association between the additive composite score and high serum uric acid by gender. Consistent with findings in the whole sample, we found that a higher additive composite score was associated with high serum uric acid in the age-adjusted logistic regression model and the multivariable model 1 in both men as well as women (although the confidence interval for an additive composite score of 1 for men could be due to chance due to the 95% confidence interval including the value of 1). With additional adjustment for clinical variables, the association was attenuated. A significant *P*-trend existed in the age and multivariable model 1 for both genders.

Similarly, Table 4 presents the association between the additive composite score of sleep variables and high serum uric acid by race/ethnicity. Here also, we found that higher additive composite score of sleep variables as well as the *P*-trend were associated with high serum uric acid in the age-, sex-adjusted and the multivariable model 1 in all major racial/ethnic groups studied, but was no longer associated with additional adjustments in the multivariable model 2. The confidence intervals included the value of 1, indicating the results may be due to chance for non-Hispanic whites, and Mexican Americans with additive composite scores of 1 in the age-, sex-adjusted model. Also, for Mexican Americans, the confidence interval included the value of 1 in the additive composite score of 1 category in multivariable model 1.

**Figure 1:**
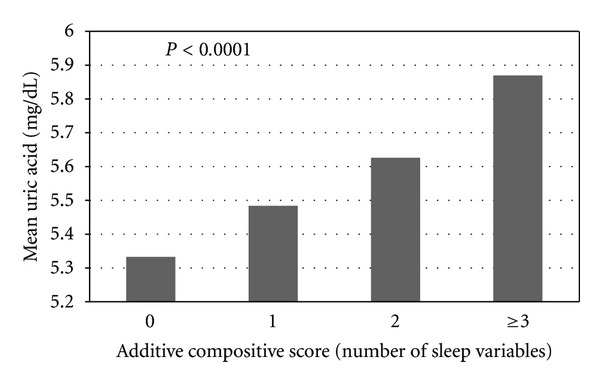
Multivariable-adjusted mean uric acid levels in relation to increasing additive composite score of sleep variables. Multivariable model adjusted for age (years), gender (male, female), education (below high school, high school, and above high school), smoking (never smoker, former smoker, and current smoker), and alcohol intake (absent, present).

Figure 1 displays the mean uric acid levels in relation to the additive composite scores of the sleep variables with the 95% confidence levels in age-, sex-adjusted model. The uric acid levels increased from a mean of 5.33 mg/dL with no sleep variables to 5.87 mg/dL with 3 or more sleep variables (*P* < 0.0001).

## 4. Discussion

In a large, national, multiethnic, cross-sectional sample of US adults, we found a significant initial association between symptoms of daytime sleepiness, snoring more than 5 nights per week and the additive composite score with high serum uric acid in an age-, gender-adjusted model, and a multivariable model adjusting for age, gender, race/ethnicity, education, smoking, alcohol intake, physical activity, and body mass index. However, the association was attenuated and not significant with the addition of clinical factors such as hypertension, depression, diabetes, and total cholesterol. 

Our initial finding of an association is significant because it has been shown that even mild hyperuricemia is associated with hypertension, diabetes, peripheral artery disease, and cardiovascular disease [8–17]. It is possible that hyperuricemia associated with the sleep variables may therefore explain part of the increased-cardiovascular disease risk seen in SDB. The exact biological mechanism underlying an association between the sleep variables and high serum uric acid is not clear. However, previous animal studies have shown that intermittent hypoxia and resultant oxygen desaturation in SDB may be associated with subsequent activation of inflammatory pathways and that serum uric acid levels are also elevated in the process [19]. In a cross-over study of 10 men exposed to 6 hours of intermittent hypoxia for 4 days, the researchers showed an increase in uric acid production during the 4 days of intermittent hypoxia and suggested that this elevation in uric acid reflects the production of reactive oxygen species through the xanthine oxidase pathway [33].

In the current study, positive associations between daytime sleepiness, snoring more than 5 nights per week, and the additive composite score with high serum uric acid were found to be initially present in the multivariable model 1, where we adjusted for lifestyle risk factors, but were found to be attenuated and no longer present in the multivariable model 2, where we additionally controlled for clinical factors such as hypertension, depression, diabetes, and total cholesterol. A similar pattern of association emerged when we performed a subgroup analysis by gender. This indicates that part of the initially observed association of the sleep variables and high serum uric acid may be mediated by the clinical factors such as hypertension, depression, diabetes, and total cholesterol. As noted earlier, there is correlation with hypertension, diabetes, BMI, and total cholesterol and these factors could be acting as effect modifiers in the association. A prospective longitudinal study is needed to explore this possibility. 

In the race/ethnicity subgroup analysis, the association persisted in the age-sex model and multivariable model 1. For non-Hispanic blacks with an additive composite score of 1, the association persisted in multivariable model 2. The exact reason for the potential racial/ethnic difference in is not clear. Non-Hispanic blacks have been reported to have higher serum uric acid levels than other racial/ethnic groups [34]. Similarly, non-Hispanic blacks have also been reported to have a higher prevalence of SDB than other racial/ethnic groups [35, 36]. Since SDB is known to be associated with increased risk of cardiovascular disease, our results indirectly suggest that hyperuricemia associated with sleep variables may be a key player in the higher CVD risk in blacks. Additional general population studies are needed to examine racial/ethnic differences in the association between sleep variables and high serum uric acid and confirm or refute our initial findings.

This study has several strengths, including the large, national sample size, high quality, standardized data collection, and the availability of information of several confounding and mediating factors. Nevertheless, there are limitations. The study design, being cross-sectional due to the nature of the NHANES sleep variables, prohibits the generation of inferential or temporal evaluations of the sleep variables and uric acid. This study used self-reported sleep variables, and there is the potential of nondifferential misclassification bias. There were 1375 participants in NHANES with missing data on sleep variables, who were excluded from the current analysis. Those who were excluded due to missing data were more significantly likely to be older, not current drinkers, and they do not exercise regularly. The exclusion of these subjects may have introduced selection bias to our results and our estimates of association may be an under- or an overestimate of the true association between sleep variables and uric acid. 

In summary, this study shows that there is an initial positive association between the sleep variables, including daytime sleepiness, snoring more than 5 nights per week, and an additive composite score of sleep variables with high serum uric acid in a national study of US adults that attenuated with adjustment for clinical factors such as diabetes mellitus, depression, total cholesterol, and hypertension. Our hypothesis-forming study suggests the need to examine the pathophysiological role of sleep risk factors with high serum uric acid levels employing polysomnography or other objective measures of sleep, and a longitudinal study design to clarify the temporal nature of this relationship.

## Figures and Tables

**Table 1:** Descriptive characteristics of the study population by categories of additive composite score^∗^.

Characteristics	Additive composite score				*P*-value
	0	1	2	≥3
Number at risk	2595	2591	1041	264	
Women (%)	59.3	46.6	39.2	39.5	<0.0001
Age (years)	43.4 ± 0.5	45.9 ± 0.6	46.0 ± 0.5	43.6 ± 0.9	<0.0001
Race/Ethnicity (%)					
Non-Hispanic whites	74.2	72.0	71.8	65.8	0.0476
Non-Hispanic blacks	8.8	9.9	12.3	16.5	<0.0001
Mexican Americans	7.6	8.9	7.2	4.6	0.2126
Others	9.4	9.3	8.8	13.1	0.6188
Education categories (%)					
Below high school	15.3	17.1	19.3	22.8	0.0003
High school	21.3	25.8	27.8	32.9	<0.0001
Above high school	63.4	57.1	52.9	44.3	<0.0001
Smoking (%)					
Never smoker	58.6	50.9	45.4	38.0	<0.0001
Former smoker	23.4	23.2	25.2	23.6	0.5163
Current smoker	17.9	25.9	29.3	38.5	<0.0001
Alcohol intake, current drinker (%)	74.4	75.3	74.8	62.2	0.0597
Moderate physical activity (%)	58.8	51.9	46.0	44.0	<0.0001
Body mass index (%)					
Normal weight	45.4	27.3	18.4	17.8	<0.0001
Overweight	33.1	36.0	32.5	26.8	0.4777
Obese	21.5	36.7	49.1	55.4	<0.0001
Depression (%)	3.6	4.1	9.9	13.4	<0.0001
Diabetes (%)	5.9	10.0	12.9	15.6	<0.0001
Total cholesterol (mg/dL)	197.9 ± 0.9	201.3 ± 1.0	202.5 ± 1.7	203.3 ± 3.5	0.0125
Uric acid (mg/dL)	5.2 ± 0.04	5.5 ± 0.03	5.7 ± 0.1	6.0 ± 0.1	<0.0001

^∗^Data presented are weighted row percentages or mean values ± standard error (SE).

**Table 2:** Association between sleep variables and hyperuricemia (defined as levels > 6.8 mg/dL in men and > 6.0 mg/dL in women).

Sleep variables	Sample size (Hyperuricemia %)	Age-, sex-adjusted OR (95% CI)	Multivariable-model 1^∗^ OR (95% CI)	Multivariable-model 2^†^ OR (95% CI)
Sleep duration (hours)				
≤5 hrs	997 (23.6)	1.15 (0.96–1.38)	1.16 (0.96–1.40)	1.01 (0.82–1.26)
6 hrs	1505 (20.8)	0.96 (0.82–1.13)	0.96 (0.82–1.14)	0.95 (0.81–1.13)
7 hrs	1871 (21.4)	1 (referent)	1 (referent)	1 (referent)
8 hrs	1710 (17.8)	0.81 (0.69–0.96)	0.82 (0.69–0.96)	0.83 (0.70–0.99)
≥9 hrs	408 (20.7)	1.05 (0.81–1.37)	1.07 (0.82–1.40)	1.15 (0.86–1.53)
*P*-trend		0.0191	0.0304	0.4799
Snoring (nights/week)				
0–2	3140 (16.2)	1 (referent)	1 (referent)	1 (referent)
3-4	1251 (20.8)	1.25 (0.98–1.58)	1.25 (0.98–1.59)	1.06 (0.83–1.35)
≥5	2100 (27.2)	1.64 (1.41–1.90)	1.69 (1.46–1.96)	1.21 (1.02–1.43)
*P*-trend		<0.0001	<0.0001	0.0278
Snorting (nights/week)				
0–2	5693 (19.8)	1 (referent)	1 (referent)	1 (referent)
3-4	417 (25.9)	1.19 (0.95–1.50)	1.20 (0.95–1.51)	1.02 (0.81–1.30)
≥5	381 (26.3)	1.26 (0.93–1.71)	1.29 (0.94–1.75)	0.89 (0.62–1.28)
*P*-trend		0.0602	0.0467	0.5622
Daytime sleepiness (times/month)				
0–15	6142 (20.4)	1 (referent)	1 (referent)	1 (referent)
≥16	349 (24.4)	1.38 (1.06–1.81)	1.42 (1.08–1.88)	1.28 (0.96–1.71)
*P*-trend		0.0167	0.0126	0.0968
Additive composite score				
0	2595 (16.4)	1 (referent)	1 (referent)	1 (referent)
1	2591 (21.8)	1.27 (1.08–1.50)	1.30 (1.11–1.54)	1.06 (0.90–1.26)
2	1041 (26.7)	1.59 (1.29–1.95)	1.63 (1.32–2.01)	1.13 (0.91–1.41)
≥3	264 (29.3)	1.88 (1.43–2.47)	1.96 (1.48–2.59)	1.27 (0.94–1.71)
*P*-trend		<0.0001	<0.0001	0.1231

^∗^Model 1: Adjusted for age (years), gender (male, female), race/ethnicity (non-Hispanic whites, non-Hispanic blacks, Mexican Americans, and others), education (below high school, high school, and above high school), smoking (never smoker, former smoker, and current smoker), and alcohol intake (absent, present) in a multivariable logistic regression model.

^†^Model 2: Additional adjusted for physical activity (moderate physical activity), body mass index (obese, nonobese), depression (absent, present), diabetes (absent, present), and total cholesterol (mg/dL) in a multivariable logistic regression model.

Abbreviations: CI: confidence interval; OR: odds ratio.

**Table 3:** Association between sleep variables and hyperuricemia, by gender.

Additive composite score	Sample size (Hyperuricemia %)	Age-adjusted OR (95% CI)	Multivariable-model 1^∗^ OR (95% CI)	Multivariable-model 2^†^ OR (95% CI)
Men				
0	1152 (23.9)	1 (referent)	1 (referent)	1 (referent)
1	1387 (26.8)	1.16 (0.95–1.41)	1.19 (0.98–1.45)	1.05 (0.88–1.26)
2	632 (29.8)	1.34 (1.01–1.77)	1.40 (1.05–1.87)	1.08 (0.81–1.44)
≥3	160 (33.0)	1.56 (1.10–2.23)	1.70 (1.18–2.45)	1.26 (0.86–1.84)
*P*-trend		0.0117	0.0039	0.3517
Women				
0	1443 (11.2)	1 (referent)	1 (referent)	1 (referent)
1	1204 (16.0)	1.41 (1.01–1.97)	1.41 (1.00–1.97)	0.99 (0.68–1.45)
2	409 (22.1)	2.12 (1.55–2.88)	2.06 (1.50–2.84)	1.21 (0.87–1.70)
≥3	104 (23.8)	2.48 (1.39–4.43)	2.31 (1.25–4.28)	1.12 (0.56–2.23)
*P*-trend		<0.0001	<0.0001	0.3906

^∗^Model 1: Adjusted for age (years), race/ethnicity (non-Hispanic whites, non-Hispanic blacks, Mexican Americans, and others), education (below high school, high school, and above high school), smoking (never smoker, former smoker, and current smoker), and alcohol intake (absent, present) in a multivariable logistic regression model.

^†^Model 2: Additional adjusted for physical activity (moderate physical activity), body mass index (obese, nonobese), depression (absent, present), diabetes (absent, present), and total cholesterol (mg/dL) in a multivariable logistic regression model.

Abbreviations: CI, confidence interval; OR, odds ratio.

**Table 4:** Association between sleep variables and hyperuricemia, by race/ethnicities^∗^.

Additive composite score	Sample size (Hyperuricemia %)	Age-, sex-adjusted OR (95% CI)	Multivariable-model 1^†^ OR (95% CI)	Multivariable-model 2^‡^ OR (95% CI)
Non-Hispanic whites				
0	1366 (17.4)	1 (referent)	1 (referent)	1 (referent)
1	1238 (22.2)	1.21 (0.99–1.47)	1.25 (1.02–1.53)	1.02 (0.83–1.26)
2	486 (26.1)	1.43 (1.12–1.83)	1.50 (1.16–1.95)	1.04 (0.80–1.35)
≥3	122 (30.3)	1.86 (1.30–2.65)	2.05 (1.38–3.03)	1.35 (0.87–2.11)
*P*-trend		<0.0001	<0.0001	0.3788
Non-Hispanic blacks				
0	484 (16.1)	1 (referent)	1 (referent)	1 (referent)
1	498 (26.0)	1.75 (1.35–2.26)	1.72 (1.32–2.24)	1.45 (1.05–1.98)
2	253 (30.9)	2.14 (1.43–3.21)	2.12 (1.42–3.17)	1.46 (0.95–2.24)
≥3	74 (30.9)	2.18 (1.23–3.88)	2.15 (1.20–3.86)	1.51 (0.79–2.91)
*P*-trend		0.0003	0.0004	0.0957
Mexican Americans				
0	459 (12.0)	1 (referent)	1 (referent)	1 (referent)
1	541 (15.4)	1.28 (0.89–1.83)	1.29 (0.89–1.88)	1.00 (0.67–1.50)
2	174 (20.5)	1.78 (1.10–2.90)	1.82 (1.10–3.02)	1.28 (0.76–2.17)
≥3	28 (26.9)	2.64 (1.01–6.88)	2.92 (1.09–7.83)	2.10 (0.68–6.54)
*P*-trend		0.0043	0.0050	0.2120

^∗^No separate analysis by other race/ethnicities due to small sample size.

^†^Model 1: Adjusted for age (years), gender (male, female), education (below high school, high school, and above high school), smoking (never smoker, former smoker, and current smoker), and alcohol intake (absent, present) in a multivariable logistic regression model.

^‡^Model 2: Additional adjusted for physical activity (moderate physical activity), body mass index (obese, nonobese), depression (absent, present), diabetes (absent, present), and total cholesterol (mg/dL) in a multivariable logistic regression model.

Abbreviations: CI: confidence interval; OR: odds ratio.
